# Swine enteric colibacillosis: Current treatment avenues and future directions

**DOI:** 10.3389/fvets.2022.981207

**Published:** 2022-10-28

**Authors:** Joana Castro, Maria Margarida Barros, Daniela Araújo, Ana Maria Campos, Ricardo Oliveira, Sónia Silva, Carina Almeida

**Affiliations:** ^1^National Institute for Agrarian and Veterinarian Research (INIAV), Vila do Conde, Portugal; ^2^LEPABE – Laboratory for Process Engineering, Environment, Biotechnology and Energy, Faculty of Engineering, University of Porto, Porto, Portugal; ^3^ALiCE – Associate Laboratory in Chemical Engineering, Faculty of Engineering, University of Porto, Porto, Portugal; ^4^Centre of Biological Engineering, Braga, Portugal; ^5^LABBELS – Associate Laboratory, Braga/Guimarães, Portugal

**Keywords:** enteric colibacillosis, post-weaning diarrhea, gut microbiome, antibiotic therapy, zinc oxide, novel antimicrobial approaches

## Abstract

Enteric colibacillosis is a common disease in nursing and weanling pigs. It is caused by the colonization of the small intestine by enterotoxigenic strains of *Escherichia coli* (ETEC) that make use of specific fimbria or pili to adhere to the absorptive epithelial cells of the jejunum and ileum. Once attached, and when both the immunological systems and the gut microbiota are poorly developed, ETEC produce one or more enterotoxins that can have local and, further on, systemic effects. These enterotoxins cause fluid and electrolytes to be secreted into the intestinal lumen of animals, which results in diarrhea, dehydration, and acidosis. From the diversity of control strategies, antibiotics and zinc oxide are the ones that have contributed more significantly to mitigating post-weaning diarrhea (PWD) economic losses. However, concerns about antibiotic resistance determined the restriction on the use of critically important antimicrobials in food-producing animals and the prohibition of their use as growth promoters. As such, it is important now to begin the transition from these preventive/control measures to other, more sustainable, approaches. This review provides a quick synopsis of the currently approved and available therapies for PWD treatment while presenting an overview of novel antimicrobial strategies that are being explored for the control and treatment of this infection, including, prebiotics, probiotics, synbiotics, organic acids, bacteriophages, spray-dried plasma, antibodies, phytogenic substances, antisense oligonucleotides, and aptamers.

## Introduction

Post-weaning period is a critical phase in the pig's life because the immune system is immature, and the sow milk removal, and consequent interruption of nutritive intake of immunoglobulin present in the milk, increases pigs' susceptibility to microbial infections, especially in the current lines of hyperprolific sows (1). As such, piglets are highly susceptible to Post-Weaning Diarrhea (PWD), a disorder that often affects pigs during the first 2 weeks of weaning and is characterized by sudden death or diarrhea and growth retardation in surviving piglets (2). PWD is one of the most serious threats to the swine industry worldwide, with episodes reaching mortality rates of 20 to 30% (2) and is associated with the proliferation of enterotoxigenic *Escherichia coli* (ETEC) in the pig intestine (3).

ETEC are gram-negative, flagellated bacilli and most pathogenic strains form smooth to mucoid colonies. Virulence factors include fimbriae, enterotoxins, endotoxins, and capsules. Fimbriae are the small hair-like structures on the bacterial surface that allow attachment to specific receptors on the surface of mucosal enterocytes of the small intestine, being crucial in the colonization process. Pathogenic strains also produce one or more enterotoxins, which are exotoxins elaborated locally in the small intestine that can have either local or systemic effects (1). There are 5 common, antigenically different fimbriae types found in pigs: F4 (K88), F5 (K99), F41, F6 (987P), and F18. The first 4 fimbriae types are responsible for mediating adhesion in neonates, while F18 is not associated with neonatal colibacillosis but is common in postweaning colibacillosis as is F4. It is also important to note that hemolysis is a common trait for pathogenic F4 and F18 isolates. Furthermore, the virulence of ETEC can also be characterized by the production of heat-labile toxins (LT), heat-stable toxin A (StA), heat-stable toxin B (StB), and verotoxin (shiga-like toxin, STX). The first three act locally interfering with electrolytes fluid increasing the fluid secretion to the lumen leading to diarrhea while verotoxin is responsible for the systemic vascular effects of edema disease (4).

As an attempt to promote health and growth performance, different approaches have been used to prevent PWD, including mostly dietary supplementation, such as prebiotics and probiotics; genetic breeding for ETEC-resistant herds; administration of growth promotors; and vaccines (5, 6). It is important to highlight that growth promoters and zinc oxide (7), have been (or are being) banned from animal husbandry. Also, antibiotics use is now more restricted because of antimicrobial resistance observed in ETEC and potential consequences for human health (1). These restrictions are strongly affecting the control of PWD (8). This makes room for innovative, antibiotic-free, intervention/control strategies that can control ETEC infections.

## Antibiotics used to treat PWD

Antibiotic therapy is required in many cases of enteric colibacillosis, being the main antimicrobials used for the treatment of enteric colibacillosis listed in Supplementary Table 1. According to Luppi, the choice of antibiotics for the treatment of PWD must consider several aspects (1): (i) the local of infection that must be located mainly in the small intestine; (ii) the empiric treatments based on the knowledge of the individual herd and local data on the resistance pattern; and (iii) the evaluation of the isolated strain's antimicrobial susceptibility. However, as an outbreak of colibacillosis frequently requires quick actions, the use of antibiotics almost always precedes the results of the resistance pattern (1, 9).

Also, it has been established that the antibiotic should be administered to all animals exhibiting signs referable to colibacillosis and sick pigs must be treated parenterally since they eat and drink very little. In practice when mortality occurs, a metaphylactic approach is applied wherein all animals in the pens are treated (1, 9–11). Of note that guidelines for the prudent use of antimicrobials in veterinary medicine (2015/C/ 299/04) published in the official journal of the European Union considered the use of metaphylaxis and stated that antimicrobial metaphylaxis should be prescribed only when there is a real need for treatment and that the veterinarian should justify and document the treatment on the basis of clinical findings on the development of a disease in herd or flock (10).

## Current measures used to prevent PWD in piglets

**Figure 1:**
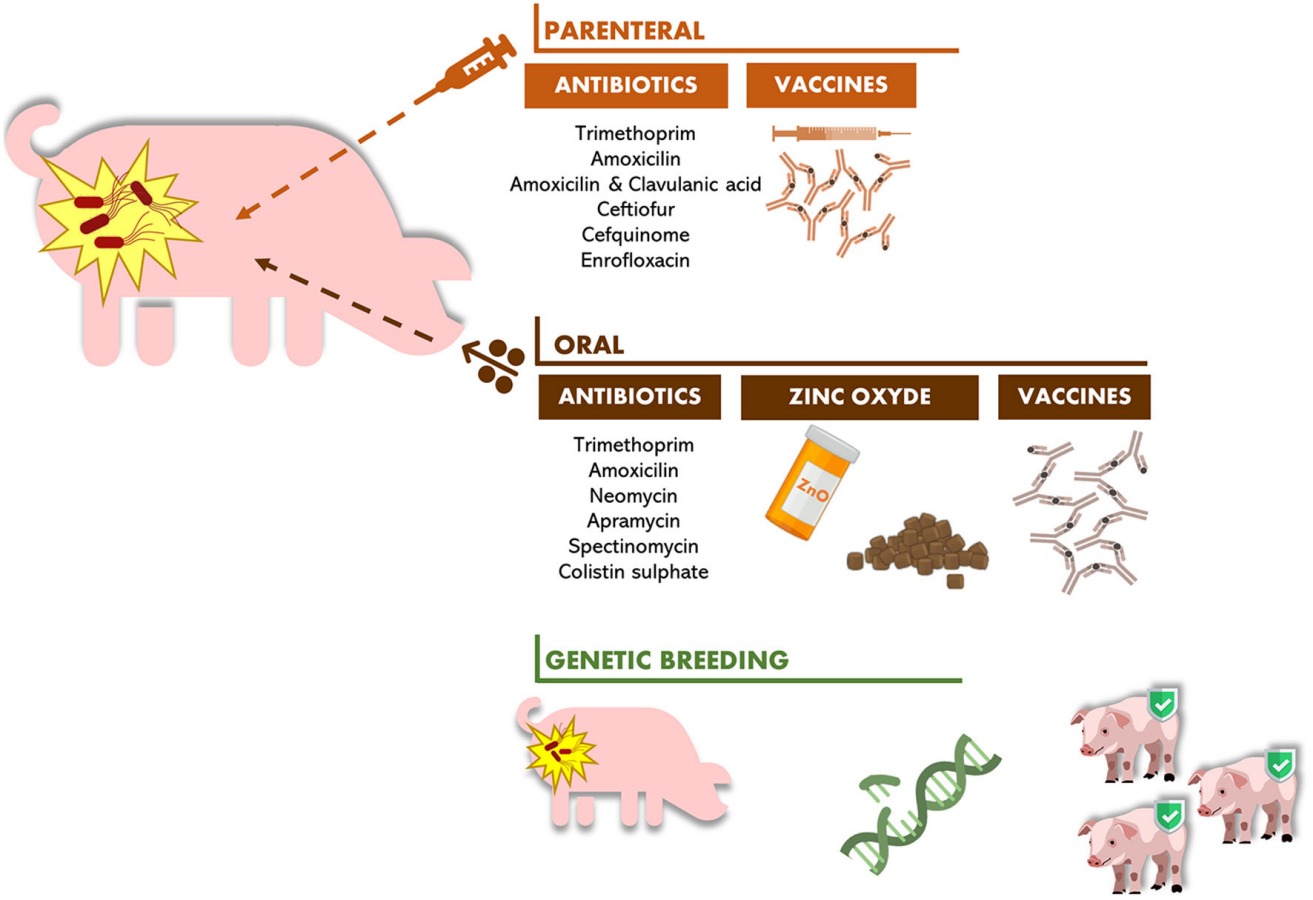
Current therapies used to control PWD in piglets.

As described above, alternatives to antibiotic therapy are some preventive approaches that have been applied to avoid the development of colibacillosis illness. Those therapies are essentially based on genetic breeding, vaccines, and zinc oxide administration, as illustrated in Figure 1.

### Genetic breeding for ETEC-resistant swine herds

The advances in molecular approaches sparked the interest of the scientific community in obtaining genetic breeding for ETEC-resistant herds. As mentioned above, the pig population presents specific receptors on the intestinal epithelial cells for the F4 and F18 fimbriae, which are the primary virulence factors carried by *E. coli* responsible for PWD. However, certain pigs do not have receptors for F4 and F18 adhesins and are thus resistant to infection by these fimbriae. Therefore, an attractive approach to preventing PWD is increasing the presence of both the F18 and F4 resistance loci in the pig population through breeding (12). Despite all the efforts, there are more insights about F18 resistance loci than about F4 resistance loci. In this sense, it was employed a molecular test for the large-scale selection of resistant pigs for breeding, using the polymerase chain reaction-restriction fragment length polymorphism (PCR-RFLP) methodology. In brief, using this approach, we can detect polymorphisms in the *FUT1* gene in a piglet, that encodes an α-(1,2) fucosyltransferase protein. It is important to highlight that *FUT1* polymorphisms are responsible for controlling the expression of the receptor for F18 fimbriated *E. coli* in the intestinal mucosa. Regarding the F4 resistance loci, by using PCR-RFLP approach (13), it is also possible to detect polymorphisms in the MUC4 gene in a piglet, that encodes for a membrane-bound-O-glycoprotein that is extensively expressed on the surface of gastrointestinal epithelial cells in which protects and lubricates the epithelial surfaces (14, 15). As such, using this approach, it was possible to select the piglets that do not present these receptors for the *E. coli* F18 and F4 adhesion (12, 16, 17).

### Vaccines

It is known that newborn pigs can establish immune reactions against mucosal antigens (18, 19). However, the newly weaned pigs need active intestinal mucosal immunization due to the lack of passive lactogenic immunity. Therefore, these vaccines can induce this protective effect by activating the mucosal immune system and antigen-specific immunoglobulins (A and M) responses (20, 21). Thus, 3 main types of vaccines have been used on pigs. Firstly, intramuscular injectable vaccines stimulate systemic immunity by increasing circulating antibodies to keep intestinal bacteria levels low enough to be non-pathogenic (21). Secondly, an oral administration of live attenuated or live wild-type non-enterotoxigenic *E. coli* strains with fimbrial adhesins can stimulate intestinal colonization by this *E. coli* by inducing the secretion of intestinal antibodies, and finally blocks the adherence of ETEC (22). For example, a single dose of a commercial vaccine (Coliprotec^®^ F4) showed a significant reduction in the incidence of diarrhea, ileum colonization by F4-ETEC, and fecal shedding of F4-ETEC after the heterologous challenge at 7 and 21 days post-vaccination (23). Lastly, the oral administration of purified fimbria results in a specific mucosal immune response in the intestines and may cause a significant decrease in fecal excretion of the pathogenic *E. coli* (21, 24). Supplementary Table 2 shows the current vaccines available in the market.

### Zinc oxide and end-of-use policies

Pig producers have been successful with weaning at an early age with limited signs of gastrointestinal disease shortly after weaning (25, 26). In fact, it has been described that feed containing between 2,400 and 3,000 ppm of zinc reduces diarrhea and mortality, and improves the growth of piglets. However, in the last decade, several studies have centered attention on heavy metals used in animal farming and possible mechanisms that could promote the spread of antibiotic resistance via co-selection (1). Furthermore, the use of zinc oxide possesses an environmental hazard, as most of the zinc is excreted with the feces and through manuring, it accumulates in soil (27).

Recently, the Committee for Medicinal Products for Veterinary Use (CVMP) of the European Medicines Agency set the referral procedure for veterinary medicinal products containing zinc oxide to be administered orally to food-producing species. The Committee adopted an opinion by consensus determining that the benefits of zinc oxide for the prevention of diarrhea in pigs do not outweigh the risks to the environment. The CVMP emphasized that there is a risk of co-selection for resistance related to the use of zinc oxide, but at the present time, that risk is not quantifiable. Accordingly, medicinal zinc oxide has been prohibited in the European Union effectively coming into force no later than June 2022 (28, 29). Consequently, there is an urgent need for adopting novel strategies for the control of PWD.

## Alternative strategies to current therapies for PWD control

The increasing evidence that the current approaches to treat/prevent PWD raise problems associated with antimicrobial resistance or the risks to the environment sparked the interest of the scientific community in exploring new eco-friendly prophylactic or antimicrobial tools. Thus, in recent years, studies of anti-enteric colibacillosis agents started to include prebiotics, probiotics, synbiotics, organic acids, phytogenic substances, bacteriophages, spray-dried plasma, antibodies, antisense oligonucleotides, and aptamers (see Table 1).

**Table 1:** Benefits and limitations of the major alternative agents for the control of post-weaning diarrhea (PWD) based on studies published since 2010.

**Agent**	**Application/Tested**	**Main results**	**Reference**
**Prebiotic**			
***In vitro***			
Anti-adhesive feedstuffs (wheat bran; casein glycomacropeptide; MOS; locust bean extract; and *Aspergillus oryzae* fermentation extract)	—Assessment of the adhesion of ETEC to the IPEC-J2 cells and analysis of the innate immune response.	—The supplementation of an alginate-derived oligosaccharide could alleviate damages exerted by ETEC infection by decreasing apoptosis.	(30)
Galacto-oligosaccharides (GOS)	—Assessment of the adhesion of *E. coli* K88 to piglet mucins.	—Neoglycans of porcine albumin conjugated with GOS partially inhibited *E. coli* K88 adhesion to piglet mucins *in vitro*.	(31)
***In vivo***			
Fructooligosaccharides (FOS)	—24 weaned pigs were divided into 3 groups: (1) control with non-challenge pigs, (2) ETEC-challenge ones, and (3) ETEC-challenge with FOS treatment.	—FOS could improve the growth performance and intestinal health in weaned pigs upon ETEC challenge.	(32)
Manno-oligosaccharide (MOS)	—32 pigs were assigned to different treatments and fed with basal or MOS diet.	—MOS can alleviate ETEC-induced injury, which was associated with suppressed inflammation and improved antioxidant capacity and intestinal epithelial functions.	(33)
**Probiotic**			
***In vitro***			
*Lactobacillus plantarum*	—Assessment of the adhesion of ETEC to IPEC-J2 cells.	—*L. plantarum* might prevent ETEC growth, inhibit ETEC adhesion to the intestinal mucosa, and activate the innate immune response to secret antimicrobial peptides.	(34)
*Pediococcus pentosaceus*	—Assessment of the adhesion of ETEC to IPEC-J2 IEC cells.	—*P. pentosaceus* decreased the adhesion of ETEC to IPEC-J2 cells.	(35)
*Enterococcus faecium, Lactobacillus rhamnosus, Bifidobacterium breve* and *Faecalibacterium prausnitzii*	—The CoMiniGut model, mimicking the small intestine of piglets, was used to evaluate the potential of 4 probiotic strains against the development of diarrhea in weaned pigs.	—This model was not representative of the high variation among pigs, so the results regarding the effect of probiotics were not clear.	(36)
*Bacillus subtilis*	—Assessment of the cytotoxicity of ETEC on IPEC-J2 IEC cells.	—It was observed that pre- or co-incubation with *B. subtilis* protected IPEC-J2 cells from ETEC-induced cytotoxicity.	(37)
*E. faecium*	—Determination the effect of the treatment with *E. faecium* on the internal redox state, paracellular permeability, IL-6 and IL-8 secretion of IPEC-J2 cells and adhesion of ETEC to IPEC-J2 IEC cells.	—*E. faecium* was able to reduce oxidative stress and paracellular permeability of IPEC-J2 cells and could inhibit the adhesion of *E. coli*	(38)
*Lactobacillus acidophilus, Lactobacillus plantarum* and *P. pentosaceus* Lactic acid bacteria (LAB)	—Determination of the antimicrobial activity of cell-free culture supernatants (CFCS) against pathogenic *E. coli*.	—CFCS from LAB can inhibit the growth of pathogenic *E. coli*.	(39)
***In vivo***			
*Pediococcus acidilactici* and *Saccharomyces cerevisiae boulardi*	—43 were assigned for this study.	—The administration of *P. acidilactici* or *S. cerevisiae boulardii* was effective in reducing ETEC attachment to the ileal mucosa, whereas the presence of *P. acidilactici* was required to modulate the expression of intestinal inflammatory cytokines in pigs challenged with ETEC.	(40)
Probiotics A: Calcium carbonate kieselguhr as anticaking agent *B. subtilis, Bacillus licheniformis* (BioPlus2B -commercial probiotic) B: Saccharose as carrier cellulose derivative as an encapsulating agent, *E. faecium* (Cylactin LBC - commercial probiotic)	—30 sows and 48 piglets of the Danbred breed were assigned to different treatments.	—No significant differences between treatment groups and controls were found concerning the immune parameters evaluated (proportion of white blood cells and their subpopulations, and serum concentration of cytokines and acute phase proteins) except for probiotic product B.	(41)
*Clostridium butyricum*	—48 weaned piglets were assigned to either a basal diet or a *C. butyricum-*supplemented diet.	—*C. butyricum* enhanced intestinal barrier function and inhibited apoptosis-associated speck-like protein-independent NLRP3 inflammasome signaling pathway.	(42)
Bacteriocins produced by *E. coli* strains	—25 weaned piglets were divided into 5 subgroups: the control without probiotic candidates and the 4 others groups were inoculated with a single probiotic candidate (582, B771, or B1172) or the combination of the 3 (cocktail).	—It was observed a significant reduction in pathogen counts for the cocktail of probiotic candidates, in comparison with the untreated control.	(43)
*L. acidophilus, L. casei, Bifidobacterium thermophilum*, and *E. faecium*	—32 newly weaned crossbred pigs were assigned to different diets.	—The multispecies probiotics enhanced growth performance by reducing intestinal inflammation, oxidative stress, and morphological damages.	(44)
*Lactiplantibacillus plantarum* and *P. acidilactici*	—72, 2-day-old, healthy neonatal piglets were recruited into the study.	—Supplementing with microencapsulated probiotics helped to improve the composition of the gut microbiota by increasing the numbers and proportions of beneficial bacteria.	(45)
*Lactobacillus casei*	—48 newborn piglets were allocated randomly to 2 groups: treated with recombinant *L. casei* and a control with a basic diet.	—The oral administration of recombinant *L. casei* favors the functional maturation of the intestinal microbiota in newborn piglets.	(46)
*Lactobacilli, Lactococci, Enterococci, Streptococci, Leuconostoc, Pediococci* and *Lactobacillus* Lactic acid bacteria (LAB)	—32 male mice were divided into 4 groups: (1) control with PBS; (2) control of the gut inflammatory model that was challenged with *E. coli*; (3) mice orally administrated with mixed LAB and (4) mice orally administrated with mixed LAB and oral challenge with *E. coli*.	—Mixed LAB strains from wild pigs exerted a beneficial effect on the host via immunomodulation of IL-6 and IL-1b against the infection of *E. coli*.	(47)
**Synbiotics**			
***In vivo***			
*E. coli* probiotic and raw potato starch (RPS)	—49, 17-day-old, weaned piglets were randomly assigned to 4 treatments: (1) positive control; (2) only probiotic; (3) only prebiotic; and (4) prebiotic and probiotic together.	—The combination of the probiotic with the RPS resulted in a reduction of diarrhea and increased microbial diversity in the gut.	(48)
Oligosaccharide lactulose and *L. plantarum*	—72 weaned pigs were assigned to different diets.	—An increase in the ileum villous height and a reduction of the pig major acute-phase protein in serum were observed. —The inclusion of the probiotic increased the number of *L. plantarum* bacteria in the ileum and colon, and it showed a trend to reduce diarrhea.	(49)
A: Inulin, *L. reuteri, L. plantarum, L. pentosus, S. cerevisiae* B: Inulin, *L. reuteri, L. plantarum, L. pentosus, L. rhamnosus S. cerevisiae* C: Inulin, *L. reuteri, L. plantarum, L. pentosus, L. rhamnosus, L. paracasei, S. cerevisiae*	— 30 sows and 48 piglets of the Danbred breed were assigned to different treatments.	—No significant differences between treatment groups and controls were found concerning the immune parameters evaluated (proportion of white blood cells and their subpopulations, and serum concentration of cytokines and acute phase proteins) except for serum immunoglobulin concentration, which was significantly increased by synbiotic products B and C.	(41)
Fermented Soy Bean Meal (FSBM) (fermented with 3 probiotic strains: *Streptococcus thermophilus, Saccharomyces cerevisiae* and *B. subtilis*)	—24 crossbred piglets were allotted into 2 diet treatments (one with FSBM and the other one without FSBM).	—Dietary FSBM replacement improved growth performance and alleviated the diarrhea of weaned piglets challenged with ETEC K88, which could be due to modulation of fecal microbiota composition and down-regulation of inflammatory cytokines production.	(50)
Inulin, immunoglobulins, vitamins, selenium, *Enterococcus faecium*, and *Saccharomyces cerevisiae*	—14 sows were assigned in this study.	—The supplementation resulted in an improvement in average daily gain. —Treated piglets had a more diverse core microbiota, with bacteria from the *Lactobacillus* genus.	(51)
**Organic acids (OA)**			
***In vitro***			
Sorbic acid, benzoic acid and hexanoic acid	—Evaluation of the susceptibility of *E. coli* K88 to selected organic acids as antimicrobial compounds.	—Benzoic acid seems to be effective against bacterial proliferation —Benzoic acid has the ability to downregulate the expression of the *E. coli* K88 major enterotoxin genes.	(52)
***In vivo***			
Two blends of organic acids and medium chain fatty acids (OM)	—A blend of organic acids and even-numbered medium-chain fatty acids and other with organic acids and odd-numbered medium-chain fatty acids were used to prevent the inflammatory response and intestinal barrier dysfunction in EHEC-infected mice.	—Both OM1 and OM2 significantly reduced the body weight loss and production of IL-6 and TNF-α induced by EHEC. —OM1 and OM2 decreased serum D-lactic concentration, bacterial transfer to the liver and spleen.	(53)
Formic/propionic acid mixture	—30 weaning piglets were submitted to 5 diets to evaluate the effect of formic and propionic acids in the diet.	—Inclusion of OA in the diet induced a decrease in rectal temperature. —The OA supplementation alleviated the inflammatory response and reduced diarrhea incidence in piglets with ETEC.	(54)
Blend of OA and cinnamaldehyde	—Evaluation of the effects of a blend of OA and cinnamaldehyde on tissue homeostasis of piglets exposed to ETEC.	— The combination of organic acid-based feed additive and cinnamaldehyde enhanced intestinal swine health.	(55)
OA and essential oils (EO) mixture (Fumaric, malic, citric, and sorbic acids, and thymol vanillin and eugenol EOs)	—24 piglets were assigned to evaluate the effects of OA/EO on growth performance, immune system, gut barrier function, nutrient absorption, and abundance of ETEC F4.	—The supplementation of OA and EO partially enhanced gut health. —The OA/EO did not significantly attenuate the induced inflammation, the reduced digestive enzyme activities, and the increased gut permeability.	(56)
**Phytogenic substances**			
***In vitro***			
Winter savory and manuka EOs	—The minimum inhibitory concentration and minimum bactericidal concentration of winter savory and manuka EOs were determined against strains of *E. coli*	—The application of EOs alone showed slight antibacterial effectiveness. —The strong action was obtained with the blends of the 2 EOs.	(57)
***In vivo***			
FRESTA^^®^^ F Wean blend of Clove (5%, Cinnamon 3%, Fenugreek 16%, Delacon)	—120 crossbred pigs were assigned to 1 of the 4 diets: control; one treatment supplemented with apramycin antibiotic (T2), other with FRESTA^®^ F (T3) and a final group with a commercial mix of organic acids (T4).	—The administration of T3 increased average daily gain. —The apparent total tract digestibility was significantly greater at week 1 with T3 and T4 and at week 6 with T3 treatment.	(58)
Commercial EO based on cinnamaldehyde and thymol	—144 weaned piglets were allotted to evaluate the EO effect on apparent nutrient digestibility, small intestinal morphology, intestinal microbiota, immune properties and antioxidant activities.	—EO supplementation increased average daily gain and the apparent digestibility of dry matter. —The *E. coli* and total anaerobes in the rectum decreased in the EO group. —The concentrations of albumin, IgA, IgG and total antioxidant capacity were higher in the EO group compared to the control group.	(59)
*ColiFit Icaps* and *Phyto Ax'Cell* *ColiFit Icaps* (trans-cinnamald ehyde, eugenol, carvacrol, thymol, and diallyl disulfure) and Phyto Ax'Cell (plant extracts and green propolis extract)	—96 piglets were allotted to assess the effect of supplementation with two-plant additives on growth performance, clinical signs, microbial analysis, inflammatory response, intestinal morphology, and ileal gene expression.	—The supplementation with *ColiFit Icaps* and with Phyto Ax'Cell enhanced the animal response to ETEC F4. —It was not observed a significant effect of the treatments on the incidence of diarrhea prevalence or fecal score.	(60)
Calcium butyrate with tannin extract	—The effect of calcium butyrate associated with tannin extract was evaluated in terms of growth performance, incidence of diarrhea, intestinal histology, immune-expression of cyclooxenase-2 and tumor necrosis factor α in piglets.	—The basal diet supplementation with butyrate plus tannin extract reduced the incidence of diarrhea in piglets compared to the control group. — The piglets showed a lower inflammatory process in the duodenum of piglets.	(61)
Phytogenic premix (caraway oil, lemon oil, clove, cinnamon, nutmeg, onion, pimento, orange peel, peppermint, and chamomile powder) with and without short and medium-chain fatty acids (SCFA and MCFA)	—The phytogenic premix with and without short and medium-chain fatty acids was evaluated in 27 weaned piglets to protect against ETEC infection.	—The piglets with a dietary supplemented with SCFA and MCFA presented a lower average feed intake and a lower frequency of diarrhea compared to the piglets supplemented with only 0.2% of phytogenic premix. —It was revealed the prevention of diarrhea in weaned piglets, through the supplementation of feed diets with phytogenic compounds plus SCFA and MCFA.	(62)
Phytogenic feed additive (bitter citrus extract; microencapsulated blend of thymol and carvacrol; mixture of bitter citrus extract, thymol and carvacrol, premixture of grape seed, grape marc extract, green tea and hops and fenugreek seed powder)	—63, 4-week-old, weaned pigs were assigned to 7 treatment groups. The growth performance, nutrient digestibility, intestinal morphology, and immune response were evaluated in weaned pigs infected with *E. coli*.	—The average daily gain and feed efficiency were increased in the phytogenic feed additives groups and the fecal score decreased. —The treatment with a mixture of bitter citrus extract, thymol and carvacrol had a higher immunoglobulin G and immunoglobulin A concentration compared to the control group.	(63)
**Bacteriophages**			
***In vitro***			
Bacteriophage EK99P-1	—The IPEC-J2/ peripheral blood mononuclear cell transwell co-culture system was used to investigate whether the modulation of barrier disruption and chemokine secretion by phage EK99P-1 in ETEC-infected IPEC-J2 would influence immune cells.	—The phage EK99P-1 seems to prevent ETEC K99-induced barrier dysfunction in IPEC-J2 and alleviated inflammation caused by ETEC infection. —Reinforcement of the intestinal barrier, such as regulation of permeability and cytokines, by phage EK99P-1 also modulates the immune cell inflammatory response.	(64)
***In vivo***			
Bacteriophage A: Basal diet +bacteriophage (0.25 g/kg) B: Basal diet +bacteriophage (0. 5 g/kg)	—30 sows and 44 pigs were assigned to different diets.	—The inclusion of bacteriophage (0.5 g/kg) treatment led to a higher *Lactobacillus* concentration compared with the basal diet.	(65)
Lytic phage CJ12 CJ12 was isolated from the sewage of a pig farm in Korea	—24 pigs weaned at 3 weeks of age were used in this experiment. Phage was mixed with feed at a ratio of 1:1.000 (0.1%).	—Pigs belonging to the phage-treated group (10^6^ PFU/g and 10^8^ PFU/g) showed more resistance to diarrhea due to ETEC infection compared to positive control on the third day after the initial challenge.	(66)
Bacteriophage Cocktail of *Salmonella typhimurium, S. enteritidis, S. cholerasuis* and *S. derby, Staphylococcus aureus, E. coli* and *Clostridium perfringens* types A and C with 10^9^ plaque-forming units per gram (pfu/g)	—200 barrows were assigned to different diets.	—Dietary increasing levels of bacteriophage linearly improved the average daily gain, average daily feed intake and apparent total tract digestibility of dry matter.	(67)
Bacteriophage Cocktail of *S. typhimurium, S. enteritidis, S. cholerasuis* and *S. derby*), *S. aureus, E. coli* and *C. perfringens* types A and C with 10^9^ plaque-forming units per gram (pfu/g)	—30 sows and 200 weaned pigs were assigned to different diets.	—Supplementation of bacteriophage in the diet improved average daily gain and gain-to-feed ratio. —Duodenum and jejunum villus heights were considerably increased.	(68)
**Spray-dried animal plasma**			
***In vivo***			
Spray-dried porcine plasma protein (SDPP)	— Weaned pigs were assigned to different diets for 14 days post-weaning, followed by a common diet to day 28 post-weaning.	—It was observed an increase in average daily weight gain and a tendency for improved average daily intake and gain-to-feed-ratio when compared with the controls during the initial 14-day period.	(69)
SDPP	—18 pairs of intrauterine growth retardation and normal birth weight weaned pigs were allotted to be fed a starter diet containing SDPP for 2 -weeks.	—SDPP diet improved bacterial diversity and increased the abundance of *Firmicutes*, but decreased the *Proteobacteria* in colonic digesta, associating with higher genera *Lactobacillus* and lower genera *Escherichia–Shigella*.	(70)
SDPP Sow feed supplemented with 0.5% or 2.5% SDPP	—452 sows were allotted to assess if spray-dried porcine plasma in peripartum feed affects sow productivity and serological immune oxidation status markers around parturition.	—Serum glutathione peroxidase activity linearly increased with increased dietary SDPP for both prepartum and postpartum sampling periods. —In the next parturition, total born pigs linearly increased, and live born pigs tended to linearly increase as the level of SDPP increased and this result was not affected by total born litter size in the first parturition.	(71)
**Specific egg yolk antibodies**			
***In vivo***			
(Hydrogel-carbon nanotubes with IgY)	—26 crossbred Landrace 2-days-old piglets were to evaluate the efficacy of hydrogel-carbon nanotubes (H-CNT) composites in piglets challenged with ETEC.	—The piglets treated with IgY encapsulated in H-CNT composites exhibited no fever or signals of diarrhea and exhibited a higher weight gain. This suggests that IgY along the gastric passage is protected by the H-CNT composites.	(72)
Chicken egg yolk immunoglobulins (IgY)	—45 piglets were allotted to analyze inflammatory profiles in jejunal and ileal mucosa according to the gene expressions of inflammatory cytokines TNF-α, IL-22, IL-6 and IL-1β.	—After *E. coli* K88 infection, in piglets treated with IgY, the intestinal inflammation induced by *E. coli* as the gene expression levels of inflammatory cytokines TNF-α, IL-22, IL-6 and IL-1β remained unchanged.	(73)
**Lactoferrin**			
***In vitro***			
Lactoferrin Bovine lactoferrin (bLF); porcine lactoferrin (pLF); ovotransferrin (ovoTF)	—A cell attachment assay was performed to evaluate the adhesion of ETEC to the small intestinal epithelium.	—bLF, ovoTF, and pLF can decrease the number of bacteria adherents to epithelial cells.	(74)
***In vivo***			
Lactoferrin Lactoferrin was obtained from *E. coli* bacteria that were DNA-modified with a bovine seed plasmid	—20 piglets were allotted to analyze the effect of lactoferrin.	—Weight gain of 1.24 Kg/week in piglets given lactoferrin, compared to control piglets which only showed a weight gain of 0.85 Kg/week. —Decrease in the number of bacteria in the treated piglets.	(75)

### Prebiotics, probiotics, synbiotics

Maintaining a healthy gut is crucial for a pig to digest and absorb dietary nutrients efficiently (76). As such, prebiotics, which are non-digestible feed ingredients positively affecting the host by stimulating the growth and/or the activity of a limited number of bacteria in the gastrointestinal microbiota (5), can be used to modulate the gut microbiota. Probiotics are defined as viable microorganisms that when ingested in sufficient amounts reach the intestine in an active state where they can exert positive effects on the host's health status (77, 78). In last, synbiotics are defined as a mixture comprising live microorganisms (probiotics) and substrate(s) (prebiotics) selectively utilized by host microorganisms that confer a health benefit on the host (79). According to Girard and colleagues, this combination improves the survival rate and favors the growth and activity of beneficial microorganisms in the gut of the piglets (51). Among the prebiotics and probiotics evaluated for the control of ETEC colonization in pigs, inulin and *Lactobacillus plantarum* are the ones that have been more systematically assessed, either individually or in combination (see Table 1). Overall, the results of the different studies indicate that these strategies can reduce ETEC colonization and relieve some symptoms. However, a few studies also have found no significant differences between the tested probiotics and the respective controls (41). This is not surprising as the effect of these approaches is most probably highly dependent on the strains, concentrations applied, animal age, and the strategies used for the delivery in the gut. Further studies are necessary to systematically evaluate the real potential of those approaches for controlling ETEC.

### Organic acids

Organic acids (OA) are considered powerful antimicrobials, which are extensively used as feed additives in pig nutrition, in particular the short-chain fatty acids (SCFAs) and medium-chain fatty acids (MCFAs) (80). Because of their antimicrobial effects, OA are able to prevent the colonization and proliferation of pathogenic bacteria (52) and their antimicrobial effects are related to their action on cellular metabolism and their ability to deplete cellular energy. Normally, a reduction in pH of the diet can happen because of the dissociation of OA, which is favorable for the growth of lactic acid bacteria (55). In contrast, the non-dissociated OA can act as an antimicrobial agent, since it can penetrate the cell wall of bacteria and interfere with normal physiology (81). In the pigs with a diet containing OA, gastrointestinal health is improved, reducing the incidence to develop diarrhea as described in Table 1.

### Phytogenic substances

Plant-derived compounds are also studied as alternative growth promoters in pigs, as is the case of phytogenic feed additives (82–84). These compounds are added to the pigs' diet, due to their positive effects on animal growth and health, since they present antibacterial, anti-inflammatory, and antioxidant properties (59). Examples of substances used as phytogenic feed additives include essential oils, herbs, and spices (see Table 1). Recent studies reported the positive results of these compounds in pig health, such as growth performance and nutrient digestibility (82). Moreover, to improve its performance, the combination of different phytogenic substances has been explored in order to study the synergistic or complementary activity on pig health.

### Bacteriophages

Bacteriophages are non-hazardous self-replicating agents that increase their numbers as they destroy specific target bacteria (85). It is noteworthy, that groundbreaking work in bacteriophage therapy addressing colibacillosis in swine was performed in the 1980s by Smith and Huggings (86–88). After 2 decades, efforts conducted by Jamalludeen and colleagues showed that the 6 tested bacteriophages exhibited prophylactic activity against diarrhea and shedding of the challenge ETEC following experimental oral infection of pigs (89). More recently, some studies on dietary supplementation of bacteriophages for *E. coli* have been conducted aiming to improve the performance, feed efficiency, and fecal microbiota in pigs as mentioned in Table 1.

### Spray-dried animal plasma

Spray-dried plasma (SDP) is a protein-rich product from the industrial fractionation of blood from healthy animals (90). To obtain SDP, blood is collected with an anticoagulant and centrifuged to separate the blood cells. Plasma is then concentrated and spray-dried under high pressure to achieve a minimum of 80°C throughout its substance. Of note that through this procedure, proteins preserve most of their biological activity (91). Multiple modes of action of SDP have been described, including either directly influencing the immune inflammatory response locally or systemically, and/or through the indirect modification of beneficial microbial populations (90). As such, in the past decades, several studies have been conducted to assess the role of SDP in swine production and calf milk replacers to improve performance, feed efficiency, and swine health (92, 93). Furthermore, some evidence suggests that SDP supplementation can modify the composition of the intestinal microbiota (92). More recently, few researchers have also conducted experiments in this field, as depicted in Table 1.

### Specific egg yolk antibodies

Chicken IgY, known as the immunoglobulin Y (IgY), is an antigen-specific antibody produced by B lymphocytes and accumulated in the yolk of chicken eggs (94). Due to its high specificity, this approach has attracted considerable attention for the treatment of gastrointestinal infections. Importantly, Wang and colleagues proved that IgY was able to significantly inhibit the growth of *E. coli* K88 by blocking the binding of *E. coli* to small intestinal mucus (73). Nevertheless, the activity of orally administered IgY can reduce rapidly under gastric conditions. Hence several nanotechnology applications, such as chitosan-alginate microcapsules, methacrylic acid copolymers, liposomes, polymeric microspheres multiple emulsification and more recently hydrogel-carbon nanotubes composites, have been used to protect IgY from hydrolysis by gastric enzymes (72), as described in Table 1.

### Porcine and bovine forms of lactoferrin

Lactoferrin (LF), an iron-binding glycoprotein, is found in the colostrum, milk, and other secretions of many mammalian species. Lactoferrin is indicated to have several physiologic functions, including regulation of iron absorption, and it has an important role in host defense, including broad-spectrum antibacterial effects (74, 95). Recently, efforts have been conducted to assess if lactoferrin can be used to control PWD, as shown in Table 1.

### Antisense oligonucleotides

An alternative antibacterial strategy to treat colibacillosis is the development of antisense oligonucleotides therapy against *E. coli* (75, 96–98). Antisense oligonucleotides (ASOs) are short, single-stranded nucleic acid sequences that are complementary to a target mRNA. They can down-regulate gene expression by binding to their target mRNA and inhibiting its translation through the creation of a steric block to ribosome binding and/or by facilitating RNase H recruitment and RNA cleavage. ASOs have been proposed to enter into cells through high-and low-binding plasma protein receptors on the cell surface, resulting in ASO compartmentalization into lysosomes and endosomes. Through a largely unknown mechanism, ASOs are released from the vesicles into the cytoplasm where they can freely move in and out of the nucleus. Upon entry into the nucleus, ASOs can bind directly to mRNA structures and prevent the formation of the 5'-mRNA cap, modulate alternative splicing, and recruit RNaseH to induce cleavage. ASOs in the cytoplasm can also bind directly to the target mRNA and sterically block the ribosomal subunits from attaching and/or running along the mRNA transcript during translation. ASOs can also be designed to directly bind to microRNA (miRNA) sequences and natural antisense transcripts (NATs), thereby prohibiting miRNAs and NATs from inhibiting their own specific mRNA targets (99). Typically, ASOs are synthesized from nucleotide analogs in order to enhance the affinity for RNA and decrease the susceptibility to cellular nucleases. A key advantage of the antisense approach is that it should be possible to rationally design an antisense oligonucleotide to target any mRNA. If the target mRNA encodes an essential protein, then the ASOs may have antibacterial properties. A number of ASOs, that target a variety of mRNAs *E. coli* genes (Supplementary Table 3), have been reported to have antibacterial activity *in vitro* and provide a proof of concept for further exploitation to test *in vivo* to control colibacillosis.

### Aptamers

Aptamers are quickly gaining significance as alternative biorecognition molecules to antibodies in targeted therapy (100). They are able to bind (i.e., diagnosis tool) and inhibit the function (i.e., therapeutic tool) of other biomolecules such as virulence factors normally associated with ETEC (i.e., toxins and fimbriae), as well as being used as carriers for other biochemical compounds (i.e., drug-delivery tool) (101). Sometimes referred to as “synthetic antibodies”, aptamers are typically single-stranded DNA or RNA oligonucleotides with defined secondary motifs (e.g., loop, stem, or G-quadruplex) that confer them complex three-dimensional structures and the ability to recognize and bind targets with exceptional binding affinity and specificity to their target (102). The aptamer-target interaction results mainly from the compatibility of physical structures and/or the stacking of chemical groups that are stabilized by hydrogen bonds, electrostatic interactions, hydrophobic effects, π-π stacking, Van der Waals forces, or combinations of these different forces (103). As a diagnostic or drug-delivery tool, aptamers can be chemically linked to other compounds (e.g., fluorophores or drugs) that will have a direct action on the target when the binding complexes are formed. As a therapeutic tool, the physicochemical binding can block receptor-binding domains of the target from interacting with receptors and, thus, neutralize their action by blocking vital proteins on the cell membrane and/or cytoplasmic proteins such as virulence factors normally associated with ETEC (i.e., toxins and fimbriae) (104). Aptamers are isolated by a repetitive interactive selection procedure known as Systematic Evolution of Ligands by EXponential enrichment (SELEX). This methodology follows a standard pattern that comprises a large pool of random sequences subjected to successive steps of (1) binding, (2) partition and (3) amplification to obtain a pool of molecules enriched for those with high affinity to the target (105). Some aptamers have been reported to be specific for *E. coli* and their virulence factors (Supplementary Table 4) providing a proof of concept for further exploration of specific aptamers for the diagnosis and treatment of colibacillosis. Aptamers for *E. coli* K88 fimbriae have been reported (106) and are of particular relevance as this is a high prevalent ETEC fimbria-type that allows adhesion to the animal gut cells. However, until now the potential of aptamers to block fimbriae-mediated adhesion in animal models has not been evaluated.

## Conclusion and future directions

Swine colibacillosis prevention and control therapies are facing a huge challenge because of the recent policies on the pharmacological dose of zinc oxide and antibiotic use. While these policies are inevitable and necessary to deal with the problem of antimicrobial resistance and environmental sustainability; the need for effective strategies to control infectious disease in pigs' production is a pressing matter. By properly addressing the virulence factors of ETEC, novel strategies will hopefully arise with more eco-friendly approaches to control PWD and more in line with the public health concerns regarding antimicrobial resistance.

## Author contributions

JC, MB, DA, AC, RO, and SS prepared the first draft of the manuscript. JC and CA defined the content of the manuscript. All authors critically reviewed and approved the final version of the article.

## Funding

Research in swine colibacillosis in CA Laboratory was supported by funding from the Fundação para a Ciência e a Tecnologia (FCT) Strategic Project Unit PTDC/CVT-CVT/4620/2021. It was also partially funded by LA/P/0045/2020 (ALiCE), UIDB/00511/2020 and UIDP/00511/2020 (LEPABE), funded by national funds through FCT/MCTES (PIDDAC), by FCT under the scope of the strategic funding of UIDB/04469/2020 unit (CEB), and by LABBELS-Associate Laboratory in Biotechnology, Bioengineering, and Microelectromechnaical Systems, LA/P/0029/2020.

## Conflict of interest

The authors declare that the research was conducted in the absence of any commercial or financial relationships that could be construed as a potential conflict of interest.

## Publisher's note

All claims expressed in this article are solely those of the authors and do not necessarily represent those of their affiliated organizations, or those of the publisher, the editors and the reviewers. Any product that may be evaluated in this article, or claim that may be made by its manufacturer, is not guaranteed or endorsed by the publisher.
