# Supplementary material for: Swine enteric colibacillosis: Current treatment avenues and future directions
Source: Front Vet Sci. 2022 Oct 28;9:981207. doi: 10.3389/fvets.2022.981207 (PMC9650617; doi:10.3389/fvets.2022.981207)
Supplement: Supplementary file 1 [file Table_1.DOCX]

Supplementary Material

Supplementary Table 1. Main antimicrobials used in treatment of swine enteric colibacillosis

| **Antimicrobial/ Prescribing category^a^** | **Administration and dosage (mg/kg body weight)** | | | | | | | | | **Adminsitration in enteric colibacillosis** |
| --- | --- | --- | --- | --- | --- | --- | --- | --- | --- | --- |
|  | **Injection** | | | | **In water** | | **In feed** | | |  |
| **TETRACYCLINE / Class One – Use with Prudence** | | | | | | | | | | |
| **Chlortetracycline** | | NA | | 22 | | | | | NA | Orally |
| **Oxytetracycline** | | 3-5 | | NA | | | | | NA | IM |
| **SULFONAMIDE/** **Class One – Use with Prudence** | | | | | | | | | | |
| Trimethoprim | 15 | | | | 30 | | 15 | | | IM and orally |
| **SYNTHETIC PENINCILLINS/ Class One/Two – Use with Prudence/Caution** | | | | | | | | | | |
| Amoxicilin | 7 | | | | 20 | | 15-20 | | | IM and orally |
| Amoxicilin plus Clavulanic acid | 7.5 + 1.75 | | | | NA | | NA | | | IM |
| **AMINOGLYCOSIDES/ Class Two – Use with Caution** | | | | | | | | | | |
| Neomycin | NA | | | | 11 | | 11 | | | Orally |
| Apramycin | NA | | | | 7.5-12.5 | | 4-8 | | | Orally |
| Spectinomycin | NA | | | | 10-50 | | 1.1-2.2 | | | Orally |
| Gentamicin | 1 | | | | 0.5+0.5+0.5 | | NA | | | IM/Orally |
| **FLUOROQUINOLONES/ Class Three – Restrict use** | | | | | | | | | | |
| Enrofloxacin | 2.5 | | | | NA | | NA | | | IM |
| **CEPHALOSPORINS/ Class Three – Restrict Use** | | | | | | | | | | |
| Ceftiofur | 3 | | | | NA | | NA | | | IM |
| Cefquinome | 1-2 | | | | NA | | NA | | | IM |
| **POLYMIXIN/ Class Three – Restrict use** | | | | | | | | | | |
| Colistin sulphate^b^ | NA | | | | 100.00 IU/Kg body weight | | 100.00 IU/Kg body weight | | | Orally |
| **QUINOXALINES^c^/Not included in prescribing category list** | | | | | | | | | | |
| Carbadox | | | NA | | | NA | | 50 | | Orally |
| Olaquindox 10% | | | NA | | | NA | | 100000/kg | | Orally |

Abbreviation: IM (intramuscular); NA (not applicable). Notes: ^a^ Prescribing guidance note for the use of antimicrobials under the cascade. Class 1 – refers to standard prescribing within responsible use guidelines. Class 2 – not to be used unless sensitivity tests or clinical experience has proven that first-choice antimicrobials are not effective. Class 3 – products of last resort; only to be used when no other options are available and supported by laboratory sensitivity tests or in extreme circumstances when all else has failed. ^b^ The use of colistin in Europe varies widely between countries. Countries with intensive livestock production can have a level of practice below 1 mg/PCU (e.g. Denmark and the UK) or much higher, up to 20 to 25 mg/PCU (Italy and Spain). Furthermore, some countries like USA and Canada never approved colistin usage in animal feed (Kumar et al., 2020). ^c^ The authorization of carbadox and olaquindox as feed additives was withdrawn in the European Union in 1999.

Supplementary Table 2. List of current vaccines available in the market used in the control of swine enteric colibacillosis

| Vaccines | Dosage | Typology of vaccine | Manufacturer |
| --- | --- | --- | --- |
| Porcilis® coli  (F4ab, F4ac, F5, F6, LT) | 2 mL | Vaccine to provide passive immunity to the progeny against *Escherichia coli* and *Clostridium perfringens* in pigs (live attenuated) | *MSD Animal Health* |
| Porcilis® 2*4*3  ((K88, K99, F41 e 987P) | 2 mL | Vaccine to provide passive immunity to the progeny against *E. coli*and *C.perfringens* in pigs (live attenuated) | *MSD Animal Health* |
| SUISENG® Coli/C  (F4ab, F4ac, F5, F6, LT) | 2 mL | Inactivated vaccine against neonatal colibacillosis and Clostridium infections in swine, in injectable suspension (live attenuated) | *HIPRA* |
| PILI SHIELD®  (K99, K88, 987P e F41) | 2 mL | Inactivated (live attenuated) | *VETFARMA* |
| SERKEL GASTRO RV®  (K88, 987P, K99, F41) | 2 mL | Inactivated (live attenuated) | *Vencofarma* |
| COLIDEX-C  (K88, K99, F41, P987, LT, Sta, VT, Hly) | 4 mLfor adults and 1 mL for piglets | Inactivated vaccine against neonatal and post-weaning diarrhea in piglets caused by *E. coli* and *Clostridium perfringens* type C. (live attenuated) | *CZ - VACCINES* |
| Enteroporc Coli  (F4ab, F4ac, F5, F6) | 2 mL | Neonatal piglet colibacillosis vaccine (recombinant, inactivated) (live attenuated) | *Ceva Salud Animal* |
| Coliprotec F4/F18  (F18ac, F4ac) | 2 mL | Live attenuated | *Prevtec microbia* |
| Entero-Vac | (100/500) dose | Avirulent live *Escherichia coli* vaccine recommended as an aid in the prevention of enteritis caused by K88 and *E. coli* in swine | *ARKO Laboratories* |
| Edema-Vac | (100/500) dose | Avirulent live culture to the prevention of edema disease caused by F18-positive *E. coli* | *ARKO Laboratories* |
| Prosystem RCE | 2 mL | Intramuscular injectable vaccine with four major *E. coli* pilus antigens (K88, K99, F41 and 987P) | *Merck Animal Health* |
| Prosystem CE | 2 mL | Intramuscular injectable vaccine with four major *E. coli* pilus antigens (K88, K99, F41 and 987P) | *Merck Animal Health* |
| Neocolipor  (F4ab (K88ab), F4ac (K88ac), F4ad (\|K88 ad) F5 (K99) F6 (987P) and F41) | 2 mL | Live attenuated vaccine that contains inactivated strains of *E. coli* | Merial |
| LitterGuard®  (K99, K88, 987P, F41) | 2 mL | Live attenuated vaccine that contains chemically inactivated strains of *E. coli.* | Zoetis |
| LitterGuard® LT-C | 2 mL | Live attenuated vaccine that contains chemically inactivated strains of *E. coli* and gives protection against beta toxin produced by *Clostridium perfringens* Type C | Zoetis |

| Genetic target | Chemistry of antisense oligonucleotide | Reference (s) |
| --- | --- | --- |
| *acpP*  (acyl carrier protein; fatty acid biosynthesis) | Peptide-peptide nucleic acid (PNA) conjugate | **(Good et al., 2001; Tan et al., 2005; Nikravesh et al., 2007)** |
|  | Phosphorodiamidate morpholino (PMO) | **(Geller et al., 2003, 2005; Deere et al., 2005)** |
|  | dendron–PNA conjugates | **(Iubatti et al., 2022)** |
| *rpoD*  *(*[RNA polymerase](https://www.sciencedirect.com/topics/biochemistry-genetics-and-molecular-biology/rna-polymerase) primary) | Conjugated peptide [nucleic acids](https://www.sciencedirect.com/topics/materials-science/nucleic-acids) (PPNAs) | **(Bai et al., 2012)** |
| *gyrA*  (DNA gyrase; DNA replication) | Peptide- Phosphorodiamidate morpholino (PMO) conjugate | **(Rao et al., 2008)** |
| *rne*  (essential endoribonuclease RNase E) | Locked nucleic acid (LNA) gapmers | **(Goddard et al., 2021)** |
| *mazEF*  (toxin-antitoxin complex) | Peptide nucleic acid (PNA) | **(Równicki et al., 2018)** |
| *hipBA*  (toxin-antitoxin complex) | Peptide nucleic acid (PNA) | **(Równicki et al., 2018)** |
| *marORAB*  (antibiotic resistance operon) | Phosphorothioate (PS) | **(White et al., 1997)** |
| *mcr-1*  (colistin resistance) | Peptide nucleic acids (PNAs) | **(Wang et al., 2020)** |
| *acrB*  (efflux pump responsible) | liposome phosphorothioate (PS)-conjugate | **(Meng et al., 2012)** |
| *ribF*  (riboflavin biosynthesis) | 2′-alkyl modification and phosphorothioate (PS) | **(Traykovska and Penchovsky, 2022)** |

**Supplementary Table 3.** Chemistry of antisense oligonucleotide used to different genetic target of *E. coli*

**Supplementary Table 4.** Aptamers used to different genetic target of *Escherichia coli*

| Aptamer | Type | Target | Reference(s) |
| --- | --- | --- | --- |
| P12-31 | DNA | Meningitis/sepsis associated *E. coli* (MNEC) | **(Marton et al., 2016)** |
| E18R, E-5, E-11, E-12, E-16, E-17, E-18, E-19, AM-6 | DNA | *E. coli* O157:H7 | **(Amraee et al., 2017; Siddiqui and Yuan, 2021)** |
| Apt B12, Apt 7, 19, 31 and 37 | DNA | *E. coli* K88 | **(Li et al., 2011; Peng et al., 2014)** |
| E1, E2, E10, E12 | DNA | *E. coli* ATCC 8739 | **(Kim et al., 2013; Chung et al., 2015; Guo et al., 2016; Jin et al., 2017)** |
| Apt 7, 19, 31 and 37 | DNA | *Shiga toxin 1 & 2 subtypes* | **(Kaur et al., 2020)** |

**References of supplementary material**

Amraee, M., Oloomi, M., Yavari, A., and Bouzari, S. (2017). DNA aptamer identification and characterization for *E. coli* O157 detection using cell based SELEX method. *Anal. Biochem.* 536, 36–44. doi:10.1016/J.AB.2017.08.005.

Bai, H., You, Y., Yan, H., Meng, J., Xue, X., Hou, Z., et al. (2012). Antisense inhibition of gene expression and growth in gram-negative bacteria by cell-penetrating peptide conjugates of peptide nucleic acids targeted to *rpoD* gene. *Biomaterials* 33, 659–667. doi:10.1016/j.biomaterials.2011.09.075.

Chung, J., Kang, J. S., Jurng, J. S., Jung, J. H., and Kim, B. C. (2015). Fast and continuous microorganism detection using aptamer-conjugated fluorescent nanoparticles on an optofluidic platform. *Biosens. Bioelectron.* 67, 303–308. doi:10.1016/J.BIOS.2014.08.039.

Deere, J., Iversen, P., and Geller, B. L. (2005). Antisense phosphorodiamidate morpholino oligomer length and target position effects on gene-specific inhibition in *Escherichia coli. Antimicrob. Agents Chemother.* 49, 249–255. doi:10.1128/AAC.49.1.249-255.2005.

Geller, B. L., Deere, J. D., Stein, D. A., Kroeker, A. D., Iversen, P. L., and Moulton, H. M. (2003). Inhibition of gene expression in *Escherichia coli* by antisense phosphorodiamidate morpholino oligomers. *Antimicrob. Agents Chemother.* 47, 3233–3239. doi:10.1128/AAC.47.10.3233.

Geller, B. L., Deere, J., Tilley, L., and Iversen, P. L. (2005). Antisense phosphorodiamidate morpholino oligomer inhibits viability of *Escherichia coli* in pure culture and in mouse peritonitis. *J. Antimicrob. Chemother.* 55, 983–988. doi:10.1093/jac/dki129.

Goddard, L. R., Mardle, C. E., Gneid, H., Ball, C. G., Gowers, D. M., Atkins, H. S., et al. (2021). An investigation into the potential of targeting *Escherichia coli* rne mRNA with locked nucleic acid (LNA) Gapmers as an antibacterial strategy. *Molecules* 26, 1–15. doi:10.3390/molecules26113414.

Good, L., Awasthi, S. K., Dryselius, R., Larsson, O., and Nielsen, P. E. (2001). Bactericidal antisense effects of peptide - PNA conjugates. *Nat. Biotechnol.* 19, 360–364. doi:10.1038/86753.

Guo, Y., Wang, Y., Liu, S., Yu, J., Wang, H., Wang, Y., et al. (2016). Label-free and highly sensitive electrochemical detection of *E. coli* based on rolling circle amplifications coupled peroxidase-mimicking DNAzyme amplification. *Biosens. Bioelectron.* 75, 315–319. doi:10.1016/J.BIOS.2015.08.031.

Iubatti, M., Gabas, I. M., Cavaco, L. M., Mood, E. H., Lim, E., Bonanno, F., et al. (2022). Antisense Peptide Nucleic Acid–Diaminobutanoic Acid Dendron Conjugates with SbmA-Independent Antimicrobial Activity against Gram-Negative Bacteria. *ACS Infect. Dis.* doi:10.1021/acsinfecdis.2c00089.

Jin, B., Wang, S., Lin, M., Jin, Y., Zhang, S., Cui, X., et al. (2017). Upconversion nanoparticles based FRET aptasensor for rapid and ultrasenstive bacteria detection. *Biosens. Bioelectron.* 90, 525–533. doi:10.1016/J.BIOS.2016.10.029.

Kaur, H., Shorie, M., and Sabherwal, P. (2020). Biolayer interferometry-SELEX for Shiga toxin antigenic-peptide aptamers & detection via chitosan-WSe_2_ aptasensor. *Biosens. Bioelectron.* 167. doi:10.1016/J.BIOS.2020.112498.

Kim, Y. S., Song, M. Y., Jurng, J., and Kim, B. C. (2013). Isolation and characterization of DNA aptamers against *Escherichia coli* using a bacterial cell-systematic evolution of ligands by exponential enrichment approach. *Anal. Biochem.* 436, 22–28. doi:10.1016/J.AB.2013.01.014.

Kumar, H., Chen, B., Kuca, K., Nepovimova, E., Kaushal, A., Nagraik, R., et al. (2020). Understanding of Colistin Usage in Food Animals and Available Detection Techniques : A Review. *Animals* 10, 1892, 1–19. doi:10.3390/ani10101892.

Li, H., Ding, X., Peng, Z., Deng, L., Wang, D., Chen, H., et al. (2011). Aptamer selection for the detection of *Escherichia coli* k88. *Can. J. Microbiol.* 57, 453–459. doi:10.1139/w11-030.

Marton, S., Cleto, F., Krieger, M. A., and Cardoso, J. (2016). Isolation of an aptamer that binds specifically to *E. coli. PLoS One* 11. doi:10.1371/journal.pone.0153637.

Meng, J., Bai, H., Jia, M., Ma, X., Hou, Z., Xue, X., et al. (2012). Restoration of antibiotic susceptibility in fluoroquinolone-resistant *Escherichia coli* by targeting *acrB* with antisense phosphorothioate oligonucleotide encapsulated in novel anion liposome. *J. Antibiot.* 65, 129–134. doi:10.1038/ja.2011.125.

Nikravesh, A., Dryselius, R., Faridani, O. R., Goh, S., Sadeghizadeh, M., Behmanesh, M., et al. (2007). Antisense PNA accumulates in *Escherichia coli* and mediates a long post-antibiotic effect. *Mol. Ther.* 15, 1537–1542. doi:10.1038/sj.mt.6300209.

Peng, Z., Ling, M., Ning, Y., and Deng, L. (2014). Rapid fluorescent detection of *Escherichia coli* K88 based on DNA aptamer library as direct and specific reporter combined with immuno-magnetic separation. *J. Fluoresc.* 24, 1159–1168. doi:10.1007/S10895-014-1396-X.

Rao, S. S., Savithri, H. S., and Raghunathan, M. (2008). Down regulation of gyrase A gene expression in *E. coli* by antisense ribozymes using RT-PCR. *Mol. Biol. Rep.* 35, 575–578. doi:10.1007/s11033-007-9126-y.

Równicki, M., Pieńko, T., Czarnecki, J., Kolanowska, M., Bartosik, D., and Trylska, J. (2018). Artificial activation of *Escherichia coli* *mazEF* and *hipBA* toxin–antitoxin systems by antisense peptide nucleic acids as an antibacterial strategy. *Front. Microbiol.* 9, 1–12. doi:10.3389/fmicb.2018.02870.

Siddiqui, S., and Yuan, J. (2021). Binding Characteristics Study of DNA based Aptamers for *E. coli* O157:H7. *Molecules* 26. doi:10.3390/MOLECULES26010204.

Tan, X. X., Actor, J. K., and Chen, Y. (2005). Peptide nucleic acid antisense oligomer as a therapeutic strategy against bacterial infection: Proof of principle using mouse intraperitoneal infection. *Antimicrob. Agents Chemother.* 49, 3203–3207. doi:10.1128/AAC.49.8.3203-3207.2005.

Traykovska, M., and Penchovsky, R. (2022). Engineering Antisense Oligonucleotides as Antibacterial Agents That Target FMN Riboswitches and Inhibit the Growth of S*taphylococcus aureus, Listeria monocytogenes*, and *Escherichia coli*. *ACS Synth. Biol.* doi:10.1021/acssynbio.2c00013.

Wang, X., Wang, Y., Ling, Z., Zhang, C., Fu, M., Wang, Y., et al. (2020). Peptide nucleic acid restores colistin susceptibility through modulation of MCR-1 expression in *Escherichia coli.* *J. Antimicrob. Chemother.* 75, 2059–2065. doi:10.1093/jac/dkaa140.

White, D. G., Maneewannakul, K., Von Hofe, E., Zillman, M., Eisenberg, W., Field, A. K., et al. (1997). Inhibition of the multiple antibiotic resistance (mar) operon in *Escherichia* *coli* by antisense DNA analogs. *Antimicrob. Agents Chemother.* 41, 2699–2704. doi:10.1128/aac.41.12.2699.
